# Risk of cancer in persons with AIDS in Italy, 1985–1998

**DOI:** 10.1038/sj.bjc.6601017

**Published:** 2003-07-01

**Authors:** L Dal Maso, S Franceschi, J Polesel, C Braga, P Piselli, E Crocetti, F Falcini, S Guzzinati, R Zanetti, M Vercelli, G Rezza

**Affiliations:** 1 Servizio di Epidemiologia e Biostatistica, Centro di Riferimento Oncologico IRCCS, via Pedemontana Occ. le 12, 33081 Aviano (PN), Italy; 2 International Agency for Reserch on Cancer, 150 Cours A. Thomas, 69372 Lyon Cedex 08, France; 3 Dipartimento di Epidemiologia, IRCCS ‘L. Spallanzani’, via Portuense, 292, 00149 Rome, Italy; 4 Registro Tumori Toscano, Epidemiologia Clinica e Descrittiva, CSPO Istituto Scientifico Regione Toscana, via S. Salvi, 12, 50135 Florence, Italy; 5 Registro Tumori della Romagna, Divisione Oncologia Medica, Istituto Oncologico Romagnolo, via Forlanini, 11, 47100 Forlì, Italy; 6 Registro Tumori del Veneto, Azienda Ospedaliera di Padova, via Gattamelata, 64, 35128 Padua, Italy; 7 Registro Tumori Piemonte, via S. Francesco da Paola, 31, 10123 Turin, Italy; 8 Dipartimento Oncologia: Biologiae Genetica, Università di Genova, Registro Tumori e Mortalità Regione Liguria, L.go Rosanna Benzi, 10, 16132 Genoa, Italy; 9 Centro Operativo AIDS, Istituto Superiore di Sanità, via Regina Elena, 299, 00161 Rome, Italy

**Keywords:** AIDS, epidemiology, haemolymphopoietic neoplasms, papillomavirus

## Abstract

A record linkage was carried out between the Italian Registry of AIDS and 19 Cancer Registries (CRs), which covered 23% of the Italian population, to estimate the overall cancer burden among persons with HIV or AIDS (PWHA) in Italy, according to various characteristics. Observed and expected numbers of cancer and standardised incidence ratios (SIRs) were assessed until 1998 in 12 104 PWHA aged 15–69 years, for a total of 60 421 person-years. Significantly increased SIRs were observed for Kaposi's sarcoma (KS, 1749-fold higher than the general population), non-Hodgkin's lymphomas (NHL, 352), and invasive cervical cancer (22). SIR was significantly elevated also for cancer of the anus (34), lung cancer (2.4), brain tumours (4.4), Hodgkin's disease (16), and leukaemias (5.3). The majority of lung and brain cancers were not histologically confirmed, and the possibility of misclassification with KS or NHL cannot be ruled out. The SIR for all non-AIDS-defining cancers was 2.2 in men and 2.5 in women. Intravenous drug users showed significantly more elevated SIRs for lung cancer (9.4), and brain tumours (6.7) than other transmission categories (SIR=1.4 and 2.3, respectively). This study confirmed increased SIRs for haemolymphopoietic neoplasms other than NHL in PWHA, although many-fold smaller than for NHL. An association with human papillomavirus-related cancers was also confirmed.

Since the mid-1990s, cancer incidence in persons with HIV or AIDS (PWHA) has been investigated by a few large population-based linkage studies of AIDS and cancer registries (CRs) in the United States (Goedert *et al*, 1998), Europe (Franceschi *et al*, 1998a), and Australia (Grulich *et al*, 2002). These studies have provided essential information on the frequency and characteristics of cancers in the course of HIV infection.

In comparison with the general population, PWHA show a thousand-fold elevated risk of developing Kaposi's sarcoma (KS) and a hundred-fold higher risk of non-Hodgkin's lymphoma (NHL) (IARC, 1996; Dal Maso *et al*, 2001c) Increases have also been reported for other cancer types, such as squamous carcinomas of the anus, invasive cervical cancer (ICC), skin, conjunctiva, and Hodgkin's disease (Franceschi *et al*, 1998a; Frisch *et al*, 2001; Grulich *et al*, 2002). Since most of these malignancies are driven by oncogenic viruses, cancer excess in PWHA is likely to be the combined effect of HIV and infections other than HIV (Boshoff and Weiss, 2002). Excesses of neoplasms, such as cancer of the lung, testis, and liver, have been reported less consistently (IARC, 1996; Frisch *et al*, 2001). Finally, in all developed countries, the introduction of the highly active antiretroviral therapies (HAART) in the mid-1990s has greatly modified the natural history of AIDS but, except for the decline in KS incidence, the effect of HAART on cancer incidence in PWHA remains unclear (International Collaboration on HIV and Cancer, 2000).

Italy offers an interesting research opportunity with respect to such issues since the AIDS epidemic has grown faster, women constitute a large proportion of PWHA (Dal Maso *et al*, 1995; Pezzotti *et al*, 1999; ENAADS, 2000), and validated AIDS surveillance programme covers the whole population while CRs cover nearly a quarter of the population.

We have updated our previous study, including twice as many person-years (Franceschi *et al*, 1998a). The purpose is to estimate the cancer burden among PWHA in Italy overall and according to time since AIDS and selected characteristics.

## MATERIALS AND METHODS

The general design of our record linkage study has been described previously (Franceschi *et al*, 1998a,1998b). In brief, notification of AIDS cases from all over Italy to the AIDS Registry (RAIDS) in Rome was initiated on a voluntary basis in 1982 and became mandatory in November 1986. At the end of 2000, a total of 47 503 AIDS cases had been reported (ISS, 2001). A linkage of RAIDS data and death certificates in 1992 suggested that under-reporting of AIDS cases in Italy was less than 10% (Conti *et al*, 1997), that is, one of the lowest percentages of under-reporting in Europe (Ajdacic-Gross *et al*, 2001).

In all, 19 independent CRs were active in Italy in the mid-1990s, covering a population of 12.7 million, corresponding to 23% of the total Italian population. They served the regions of Romagna, Friuli-Venezia Giulia, Umbria, and part of Veneto region, the municipality of Turin, the provinces of Genoa, Biella, Varese, Parma, Modena, Ferrara, Macerata, Florence and Prato, Sassari, Trento, Bolzano (Alto Adige), Ragusa, Latina, and part of the province of Naples (Zanetti *et al*, 2002; Parkin *et al*, 2002). Cancer Registeries varied both in size, ranging from approximately 190 000 to nearly 1.9 million of population covered, and in number of registration years available. Routine indicators of data completeness and quality in Italian CRs were satisfactory (Parkin *et al*, in press,^1997^; Zanetti *et al*, 2002).

An ‘*ad hoc*’ software application was developed to perform the record linkage procedure (Dal Maso *et al*, 2001c). Briefly, records from RAIDS and CRs were linked by last and first name, and by date of birth. Satisfaction of name–date algorithm required: (a) that the records were identical for at least one critical field, and (b) that the other two critical fields, if not identical, differed only in prescribed ways. Since the system operated under procedures that removed all personal identifiers, the staffs of each type of registry were blinded to which persons had been linked.

The present study was restricted to people who: (1) were aged between 15 and 69 years at the time of AIDS diagnosis; (2) reported a legal residence in areas covered by CRs; (3) were diagnosed with cancer in periods deemed complete at both registries (i.e. in most instances through to the end of 1998); and (4) were diagnosed with AIDS after 1985, since no cancer case among PWHA had been reported earlier.

AIDS-defining cancers (i.e. KS, NHL and, since 1993, ICC) are also reported to RAIDS, but only notification of neoplasms to CRs were included in the present analysis for the sake of comparability with non-AIDS defining cancers (NADC). *In situ* carcinoma of the cervix and other preneoplastic lesions (e.g. behaviour code 0–2) (WHO, 1990) were excluded from the present analysis because of incomplete reporting in CRs.

Cancers at CRs were identified according to International Classification of Diseases, 9th revision (WHO, 1977) and International Classification of Diseases for Oncology (ICDO) (WHO, 1990). Cases were subsequently recoded according to International Classification of Disease, 10th revision (WHO, 1992). Cancers were further subdivided according to whether histological, haematological, or cytological confirmation (henceforth referred to as histological confirmation) was available or diagnosis had been made otherwise (i.e. clinical, instrumental diagnosis, etc.). Cancer Registry coordinators reviewed all records on histological type and site of cancers.

When an AIDS-defining cancer was mentioned in both RAIDS and CR, date of cancer diagnosis was defined as the earliest one. When KS, NHL, or ICC (after 1993) was reported in the CR up to 5 years prior to the date of AIDS diagnosis in RAIDS, the date of AIDS onset was backdated.

Person-years at risk were computed only between 5 years prior to AIDS diagnosis (in order to exclude cancer diagnosis which may have occurred before HIV infection) and date of death or 3.5 years after AIDS diagnosis, whichever occurred earlier, to reduce inaccuracies from losses at follow-up. This interval was left- or right-censored if no complete CR data were available in the corresponding years. Expected numbers of different cancer sites or types were computed in each CR from sex- and age-specific (5 years) incidence rates (Parkin *et al*, 2002,^1997^,^1992^) in four periods: early pre-AIDS (from −60 to −25 months), late pre-AIDS (from −24 to −7 months), AIDS period (from −6 to +3 months), and post-AIDS period (from +4 to +42 months). Observed numbers of cancer in PWHA were compared to expected numbers by means of standardised incidence ratios (SIRs). Corresponding 95% confidence intervals (CI) were computed using the Poisson distribution (Breslow and Day, 1987).

## RESULTS

Overall, 12 104 AIDS cases (78% men and 22% women) were reported in areas covered by one of the 19 CRs (Table 1

**Table 1:** AIDS diagnoses and linked cases by type of cancer in 19 Italian areas covered by Cancer Registries (CRs)

		**Population**		**AIDS cases in CR areas**	**Linked cases^a^**			
**CR**	**Complete period**	**(× 1000)**	**Cancer cases**	**(1982–2000)**	**KS**	**NHL**	**ICC**	**Other cancers**
Alto Adige	1995–97	453	8107	196	4	2	0	3
Biella	1995–98	190	5782	253	3	6	1	1
Ferrara	1989–98	361	32 749	379	14	18	0	3
Florence	1985–97	1071	97 506	1050	100	58	1	29
FVG	1995–98	1186	40 002	379	6	4	0	1
Genoa	1985–96	679	76 746	1566	59	51	4	22
Latina	1983–98	527	21 630	337	5	9	1	1
Macerata	1991–97	358	12 773	112	6	2	1	0
Modena	1988–98	620	42 681	543	32	21	2	6
Naples	1996–98	525	4655	94	1	2	0	0
Parma	1982–98	391	27 745	308	17	13	0	7
Ragusa	1982–97	313	16 892	53	2	1	0	1
Romagna	1985–97	433	71 553	1485	63	64	2	22
Sassari	1992–97	468	11 883	302	7	12	0	3
Trento	1995–98	455	11 756	284	5	8	0	4
Turin	1985–98	963	85 751	1106	83	37	1	25
Umbria	1994–97	911	27 745	372	14	19	0	4
Varese	1982–97	789	54 400	1462	39	66	3	14
Veneto	1987–96	1890	1 21 347	1823	65	56	2	23
								
**Total**		12 583	6 98 380	12 104	525	449	18	170

KS=Kaposi's sarcoma; NHL=non-Hodgkin lymphoma; ICC=invasive cervical cancer; FVG=Friuli-Venezia Giulia.

a Cancers notified at CRs in people with AIDS, age 15–69 years, between 1985 and 1998 from 5 years prior to 3.5 years after an AIDS diagnosis.

) for a total of 60 421 person-years (Table 2

**Table 2:** Observed (Obs) and expected (Exp) numbers of cancers in person HIV or with AIDS, SIR, and corresponding 95% CI by time of diagnosis and overall. Italy, 1985–1998

	**60–25 months before AIDS**			**24–7 months before AIDS**			**6 months before to 3 months after AIDS^a^**			**4–42 months after AIDS**			**Total**			
ICD10, cancer type or site	**Obs**	**SIR**	**(95% CI)**	**Obs**	**SIR**	**(95% CI)**	**Obs**	**SIR**	**(95% CI)**	**Obs**	**SIR**	**(95% CI)**	**Obs**	**Exp**	**SIR**	**(95% CI)**
*Person years*	*27 882*			*14 652*			*7491*			*10 397*			*60 421*			
C46, KS							406	6667	(6034–7348)	119	497	(412–595)	525	0.30	1749	(1602–1905)
C82–C85, NHL							322	1229	(1098–1371)	127	125	(105–149)	449	1.27	352	(320–386)
C53, ICC	1	3.0	(0.0–17.5)	2	9.6	(0.9–35.4)	14	121.2	(66–204)	1	5.8	(0.0–33.1)	18	0.82	21.8	(12.9–34.6)
C16, Stomach	1	0.6	(0.0–3.7)	1	1.1	(0.0–6.2)	4	8.1	(2.1–21.1)	2	3.3	(0.3–12.2)	8	3.56	2.2	(1.0–4.4)
C18, Colon	0	0	—	2	1.8	(0.2–6.6)	1	1.6	(0.0–9.4)	1	1.3	(0.0–7.5)	4	4.21	0.9	(0.2–2.5)
C20, Rectum	0	0	—	1	1.7	(0.0–10.0)	2	6.5	(0.6–23.9)	2	5.2	(0.5–19.0)	5	2.18	2.3	(0.7–5.4)
C21, Anus	3	44.4	(8.4–131.4)	0	0	—	1	38.5	(0.0–220.9)	2	51.5	(4.9–189.2)	6	0.18	33.6	(12.1–73.6)
C22, Liver	0	0	—	0	0	—	1	4.3	(0.0–24.6)	2	6.9	(0.7–25.5)	3	1.58	1.9	(0.4–5.6)
C25, Pancreas	0	0	—	0	0	—	1	5.5	(0.0–31.4)	1	4.4	(0.0–25.1)	2	1.27	1.6	(0.1–5.8)
C32, Larynx	0	0	—	0	0	—	1	3.7	(0.0–21.0)	1	3.0	(0.0–17.3)	2	1.95	1.0	(0.1–3.8)
C33–C34, Trachea and lung	1	0.3	(0.0–1.5)	0	0	—	13	10.3	(5.5–17.7)	8	5.4	(2.3–10.8)	22	9.02	2.4	(1.5–3.7)
C43, Melanoma	0	0	—	1	1.1	(0.0–6.1)	2	3.9	(0.4–14.3)	0	0	—	3	3.72	0.8	(0.2–2.4)
C44, Skin	3	0.8	(0.2–2.4)	5	2.1	(0.7–4.9)	3	2.3	(0.4–6.7)	3	1.7	(0.3–4.9)	14	9.28	1.5	(0.8–2.5)
C50, Breast^b^	0	0	—	3	2.6	(0.5–7.8)	0	0	—	0	0	—	3	4.43	0.7	(0.1–2.0)
C56, Ovary	0	0	—	0	0	—	3	33.1	(6.2–98.1)	0	0	—	3	0.68	4.4	(0.8–13.2)
C61, Prostate	0	0	—	1	2.1	(0.0–11.8)	0	0	—	1	3.1	(0.0–17.6)	2	1.72	1.2	(0.1–4.3)
C62, Testis	0	0	—	4	4.3	(1.1–11.2)	0	0	—	0	0	—	4	3.73	1.1	(0.3–2.8)
C64, Kidney	1	0.9	(0.0–5.0)	1	1.4	(0.0–8.0)	1	2.6	(0.0–14.8)	0	0	—	3	2.75	1.1	(0.2–3.2)
C67, Bladder	0	0	—	1	0.8	(0.0–4.5)	0	0	—	1	1.2	(0.0–7.0)	2	4.80	0.4	(0.0–1.5)
C70–C72, Brain	0	0	—	0	0	—	8	24.3	(10.4–48.0)	3	6.6	(1.2–19.5)	11	2.48	4.4	(2.2–8.0)
C81, Hodgkin's disease	6	4.6	(1.6–10.0)	12	17.9	(9.2–31.4)	19	56.3	(33.8–88.1)	8	17.6	(7.5–34.8)	45	2.77	16.2	(11.8–21.7)
C90, Myeloma	0	0	—	1	6.0	(0.0–34.5)	0	0	—	2	16.6	(1.6–61.0)	3	0.62	4.8	(0.9–14.3)
C91–C95, Leukaemia, all	1	0.9	(0.0–5.4)	3	4.9	(0.9–14.4)	6	18.5	(6.7–40.5)	3	6.9	(1.3–20.4)	13	2.44	5.3	(2.8–9.2)
*C91, Leukaemia, lymphoid*	*1*	*2.7*	*(0.0–15.2)*	*0*	*0*	—	*4*	*34.0*	*(8.8–87.8)*	*0*	*0*	—	*5*	*0.87*	*5.7*	*(1.8–13.5)*
*C92, Leukaemia, myeloid*	*0*	*0*	—	*2*	*5.7*	*(0.5–21.0)*	*1*	*5.4*	*(0.0–30.9)*	*2*	*7.9*	*(0.7–29.2)*	*5*	*1.39*	*3.6*	*(1.1–8.4)*
																
All non-AIDS defining cancer^c^	18	0.6	(0.3–0.9)	39	2.0	(1.4–2.8)	68	6.6	(5.1–8.4)	45	3.3	(2.4–4.5)	170	74.48	2.3	(2.0–2.7)

SIR=Standardized Incidence Ratios; CI=Confidence Intervals; KS=Kaposi's Sacroma; NHL=Non-Hodgkin Lymphoma; ICC=Invasive Cervical Cancer.

a For KS and NHL: from AIDS to 3 months after.

b Females only.

c It includes neoplasms excluding KS, NHL and ICC plus one of each: lip; nasopharynx; digestive organs, not otherwise specified; nasal cavity; mediastinum; connective tissue; retroperitoneum; vulva; corpus uteri; uterus, not otherwise specified; penis; endocrine glands; and unknown primary site.

). In all, 70% of person-years referred to the periods prior to AIDS diagnosis. The majority of PWHA enrolled in this study were intravenous drug users (IDUs, 62%), and the median age was 33 years. Between 5 years before AIDS diagnosis and 3.5 years thereafter, 170 cancers other than KS, NHL, and ICC were identified (Table 1). Using only cancer information from CRs, the numbers were 525 for KS, 449 for NHL, and 18 for ICC.

Table 2 shows observed and expected numbers and corresponding SIRs for cancer sites or types with at least two cases observed. As expected, high SIRs were found for KS (1749; 95% CI: 1602–1905), NHL (352; 95% CI: 320–386), and, to a lesser extent, ICC (22; 95% CI: 13–35). All ICC were squamous-cell carcinomas.

The combination of NADC showed a SIR of 2.3 (95% CI: 2.0–2.7). Significantly elevated SIRs were seen for cancer of the anus (34; 95% CI: 12–74), lung (2.4; 95% CI: 1.5–3.7), brain (4.4; 95% CI: 2.2–8.0), Hodgkin's disease (HD) (16; 95% CI: 12–22), and leukaemias (5.3; 95% CI: 2.8–9.2). In respect to time of cancer diagnosis, all sites and types showed the highest SIR in the AIDS period and a compensatory fall in SIR after AIDS. Risk excess before AIDS was seen for ICC and cancer of the anus, and for HD and leukaemias.

Anal cancers (six cases) included five squamous-cell carcinomas and one cloacogenic carcinoma.

Only 12 out of 22 lung cancers were histologically confirmed, including five squamous-cell carcinoma, two adenocarcinoma, and one each of five other histologies. In four out of 10 nonhistologically confirmed lung cancer cases, lung lesions of infectious origin, such as pneumocystis carinii pneumonia, were reported at RAIDS. None of the 11 brain cancers were histologically confirmed, eight had occurred in the AIDS period, and three had a concurrent diagnosis of cerebral toxoplasmosis or HIV encephalopathy at RAIDS. The proportions of histologically confirmed cancers among PWHA (79%) were similar to the proportion in the general population of the same CRs (80%). Restricting the analysis to cancers with histological confirmation, the SIR was 1.7 (95% CI: 1.4–2.1) for the combination of all NADC. The greater reduction in SIR, after the exclusion of unconfirmed cases, was noted for cancer of the lung (SIR=1.3) and brain (SIR=0.0) (not shown).

Among 45 HDs, the histological type was mixed cellularity in 19 subjects and nodular sclerosis in 13. The other HDs were of lymphocytic predominance (one), lymphocytic depletion (three), and unspecified type (nine).

The cancer sites or types that showed a significant excess in PWHA or were observed in more than 10 individuals were re-examined in separate strata of gender, age, and HIV-exposure category (Table 3

**Table 3:** Observed cases (Obs) of selected cancers, SIR and corresponding 95% CI among people with HIV or AIDS by gender, age group, and HIV exposure category, Italy, 1985–1998

	**Gender**				**Age group**				**HIV exposure category**			
	**Men**		**Women**		**15–34**		**35–69**		**IDU**		**Other**	
		**SIR**		**SIR**		**SIR**		**SIR**		**SIR**		**SIR**
**ICD10–cancer type or site**	**Obs**	**(95% CI)**	**Obs**	**(95% CI)**	**Obs**	**(95% CI)**	**Obs**	**(95% CI)**	**Obs**	**(95% CI)**	**Obs**	**(95% CI)**
C46: KS	483	1696	42	2717	228	1218	297	2628	115	557	410^a^	4369
		(1548–1854)		(1957–3675)		(1065–1387)		(2337–2945)		(460–669)		(3956–4813)
C82–C85: NHL	350	321	99	538	268	417	181	286	247	335	202	376
		(288–356)		(437–655)		(369–470)		(246–331)		(294–379)		(326–432)
C53: ICC	—	—	18	21.8	8	23.4	10	20.7	11	24.0	7	19.1
		—		(12.9–34.6)		(10.0–46.4)		(9.9–38.2)		(11.9–43.1)		(7.6–39.6)
C21: anus	5	35.1	1	27.6	1	31.3	5	34.1	2	33.6	4^b^	33.6
		(11.1–82.5)		(0.0–158.3)		(0.0–179.3)		(10.8–80.2)		(3.2–123.6)		(8.7–86.8)
C33, C34: trachea and lung	19	2.2	3	8.7	6	19.4	16	1.8	11	9.4	11	1.4
		(1.3–3.4)		(1.6–25.9)		(7.0–42.4)		(1.0–3.0)		(4.6–16.8)		(0.7–2.5)
C44: skin (nonmelanoma)	13	1.7	1	0.7	0	0	14	1.9	1	0.4	13	2.0
		(0.9–2.8)		(0.0–4.0)		—		(1.0–3.1)		(0.0–2.0)		(1.1–3.5)
C70–C73: brain	8	3.8	3	8.5	6	6.4	5	3.2	8	6.7	3	2.3
		(1.6–7.4)		(1.6–25.3)		(2.3–14.1)		(1.0–7.6)		(2.9–13.2)		(0.4–6.9)
C81: Hodgkin's disease	36	16.7	9	14.6	33	17.9	12	12.9	34	18.1	11	12.3
		(11.7–23.1)		(6.6–27.9)		(12.3–25.2)		(6.7–22.7)		(12.5–25.3)		(6.1–22.1)
C91–C95: leukaemias	12	5.7	1	2.9	8	8.4	5	3.4	5	4.3	8	6.3
		(2.9–10.1)		(0.0–16.7)		(3.6–16.7)		(1.1–7.9)		(1.3–10.0)		(2.7–12.6)
												
All non-AIDS defining	135	2.2	35	2.5	74	4.6	96	1.6	87	3.6	83	1.7
Cancers		(1.9–2.7)		(1.7–3.4)		(3.6–5.8)		(1.3–2.0)		(2.9–4.4)		(1.3–2.1)

SIR=Standardized Incidence Ratios; CI=Confidence Intervals; KS=Kaposi's Sacroma; NHL=Non-Hodgkin Lymphoma; ICC=Invasive Cervical Cancer. IDU=Intravenous drug user.

a 310 cases among homosexual and bisexual men, SIR=6066, 95% CI: 5409–6780.

b Three cases among homosexual and bisexual men, SIR=56.4, 95% CI: 10.6–167.0.

). Standardised incidence ratios were higher in women than in men for KS (2717 *vs* 1696, respectively), NHL (538 *vs* 321), and cancer of the lung (8.7 *vs* 2.2). For the combination of all NADS, no difference emerged between genders (SIR=2.2 and 2.5 in men and women, respectively). The age distribution varied by cancer type: median age was 37 years for KS (range: 19–68), 33 years for NHL (21–68), 35 years for ICC (37–48), 42 years for anal cancer (34–55), 39 years for lung cancer (23–68), 50 years for non-melanomatous skin cancer (36–69), 31 years for brain cancer (28–58), 32 years for HD (19–43), and 32 years for leukaemias (21–67). Standardised incidence ratios tended to be higher in the 15–34-year age group than in the 35–69-year age group for all cancer sites, except for KS and cancers of the cervix uteri and anus. Standardised incidence ratios were also higher among IDUs than other HIV exposure category for all cancer types except for KS and leukaemias. Standardised incidence ratio for KS and anal cancer was particularly high among homosexual and bisexual men (6066 and 56, respectively).

The risk of NADCs in PWHA at or after AIDS diagnosis (patients who were likely to take advantage of HAART) did not diminish between pre-HAART (SIR=4.7) and post-HAART period (SIR=5.7). Moreover, no statistically significant change emerged for any specific cancer site or type of NADCs (data not shown).

## DISCUSSION

Cancers of the anus, brain, lung, HD, and leukaemias, in addition to AIDS-defining cancers (KS, NHL, and ICC), have been found in our study to be significantly increased in PWHA.

As in previous studies (Franceschi *et al*, 1998a; Sitas *et al*, 2000; Grulich *et al*, 2002; Frisch *et al*, 2001), the group of NADCs, which showed the most consistent excess in PWHA was lymphohaematopoietic cancers. HD risk, among PWHA, was more than 10-fold increased in every strata of gender, age, and HIV-exposure category. An association between HD and HIV infection is, hence, well established (Dal Maso and Franceschi, 2003). An excess of leukaemias and myeloma is also suggested by our present findings.

Histological confirmation was available for almost all HDs (43 out of 45), myelomas (three out of three), and leukaemias (10 out of 13) in our study. A misdiagnosis of NHL as other haemolymphopoietic neoplasms cannot be totally ruled out because the classification of haemolymphopoietic neoplasms in PWHA is especially difficult (Carbone, 2002). Some leukaemias might be leukaemoid transformations of AIDS-related NHL. The SIR for lymphoid leukaemia was not significantly greater than the one for myeloid leukaemia, in agreement with the findings by Frisch *et al* (2001) in the United States.

As in previous reports (Andrieu *et al*, 1993; Serraino *et al*, 1993), the most common subtype of HD in PWHA was mixed cellularity type. Nodular sclerosis type (i.e. the commonest HD type in the general population of an age comparable to PWHA) was relatively rare. Besides being more aggressive and more frequently involving bone marrow, HD in PWHA seems to be associated with EBV more often than in the general population (Tirelli *et al*, 2000).

Other cancers whose SIRs were found to be consistently elevated in PWHA are those associated with HPV infection (IARC, 1995). In contrast to KS and NHL, ICC has been an AIDS defining disease only since 1993 (Ancelle Park, 1993). We were, therefore, able to observe some ICC cases prior to AIDS and noticed that, although SIR peaked in the AIDS period as for all other sites and types, some excess was present years before AIDS. A similar time pattern was found for anal cancer, whose overall SIR (34) was well comparable to the one seen for ICC. Three additional cases of cancer of the vulva, penis, and uterus (not otherwise specified) further support the possibility that HIV-induced immune impairment facilitates the persistence of HPV infection (Palefsky *et al*, 1999; Ahdieh *et al*, 2000): progression into pre-malignant (Sun *et al*, 1997) and, ultimately, in the lack of early detection, invasive cancer. As suggested also by studies where preinvasive lesions of the cervix and anogenital tract were included (Frisch *et al*, 2000), the control of HPV infections seems to be impaired in HIV-positive women and men years before a diagnosis of AIDS. Early events in HPV carcinogenesis are probably affected to a greater extent than late ones (i.e. invasiveness). The SIR for cancer of the cervix and anus was marginally greater but not restricted to specific HIV exposure categories (i.e. IDUs and homosexual and bisexual men, respectively). Since HPV and HIV share a sexual route of transmission, it has been considered difficult to disentangle their independent contribution to the increase in risk of anogenital cancer in PWHA (Mandelblatt *et al*, 1999). A majority of sexually active women (Woodman *et al*, 2001) and men (Franceschi *et al*, 2002), however, at some point in their lifetime are infected by HPV. Therefore, factors that enhance the probability of HPV infection becoming persistent and progress into premalignant and malignant lesions are crucial (IARC 1995). In particular, a lack of cytotoxic T-lymphocyte to the early (E) oncoprotein six antigen is associated with persistence of HPV infection (Tindle, 2002).

Nonmelanomatous skin cancer showed a three-fold excess in PWHA in our previous report (Franceschi *et al*, 1998a), whereas a somewhat lower SIR (1.5; 95% CI: 0.8–2.5) emerged from our present update. Misclassification with KS seems unlikely, since all nonmelanomatous skin cancers in PWHA were histologically confirmed (seven squamous-cell and seven basal-cell carcinomas). The report of nonmelanomatous skin cancer to CRs is, however, incomplete (Levi *et al*, 1995; Parkin *et al*, 1997) and the corresponding SIR must be interpreted cautiously. No data on nonmelanomatous skin cancer are available from North American (Frisch *et al*, 2001) and Australian (Grulich *et al*, 2002) record linkage studies. Gallagher *et al* (2001) reported a 10-fold increased SIR of cancer of the lip in New York State and several authors (Frisch *et al*, 2001; Grulich *et al*, 2002) showed a two- to- four-fold increased risk. Interestingly, skin cancer in our study affected more frequently older PWHA than any other NADC.

Cancers of the lung and brain are found to be consistently increased among PWHA, but masses of non-neoplastic origin or attributable to NHL are common in the lung and brain at AIDS diagnosis. These masses may be misdiagnosed as cancer of the lung or brain, particularly in the absence of histological confirmation. Interestingly, lung cancer showed the most marked variation by HIV transmission group among NADCs. As in Serraino *et al* (1997),(2000), lung cancer seemed restricted to IDUs, among whom smoking levels are much higher than in the general population.

Hepatocellular carcinoma is another cancer type aetiologically related to viruses (i.e. hepatitis B and C viruses) that, in turn, share the same route of transmission of HIV and have a high prevalence in PWHA (Smukler and Ratner, 2002). However, our present study showed only a moderate excess (SIR=1.9) of HCC in agreement with other authors (Frisch *et al*, 2001; Gallagher *et al*, 2001; Grulich *et al*, 2002). It is possible that, until recently, PWHA have not survived long enough to allow the carcinogenic effect of hepatitis viruses to manifest (Deuffic *et al*, 1999).

Standardised incidence ratios were above unity for a few additional cancer types (i.e. stomach, rectum, and ovary), but corresponding CI were broad, due to the relatively small number of cases observed.

No reduction in the SIR of NADCs emerged in Italy after the introduction of HAART. Our present study is based, however, on too short a period after HAART to allow any conclusion.

The validity of cohorts defined by AIDS diagnosis, as in record linkage studies, to estimate risks of NADCs before AIDS has been questioned (Goedert *et al*, 1998; International Collaboration on HIV and Cancer, 2000). Li *et al* (2002), however, have recently demonstrated that in Australia one cohort based on AIDS diagnosis and two based on HIV diagnosis provided consistent SIRs for the 10 commonest NADCs.

Since a large number of NADCs occurs before AIDS, the major strength of such methodology is represented by the access to a large number of person-years of observation both before and after AIDS (Franceschi *et al*, 1998b; Goedert *et al*, 1998). The number of PWHA and of cancer diagnoses may be underestimated on account of incompleteness of reporting to either AIDS or CRs or of missed linkages. However, the completeness of RAIDS and Italian CRs had been shown to be satisfactory and the linkage procedures we used had been validated (Conti *et al*, 1997; Ajdacic-Gross *et al*, 2001; Dal Maso *et al*, 2001a). The problem of migration of PWHA out of CR areas should be less severe in Italy than elsewhere, as population mobility is comparatively low and HIV and cancer treatments are available free of charge in all Italian regions (Franceschi *et al*, 1998b). As already reported (Biggar *et al*, 1996), SIRs for all cancer sites or types are exaggerated in the months immediately before and after AIDS by an increased medical surveillance and some cancers would have been otherwise found later. Our present SIRs in the post-AIDS period are therefore underestimates and this hampers the evaluation of trends in cancer risk by immune status. Cancer excesses were also found, however, prior to AIDS diagnosis, for ICC, cancer of the anus, and HD.

## Appendix

Cancer and AIDS Registry Linkage Study: Diego Serraino (IRCCS ‘L. Spallanzani’, Rome); Gary Clifford (IARC Lyon, France); Eugenio Paci (Registro Tumori Toscano, Florence); Rosa Vattiato (Registro Tumori della Romagna, Forlì); Anna Rita Fiore (Registro Tumori del Veneto, Padova); Silvia Patriarca (Registro Tumori Piemonte, Torino); Claudia Casella (Registro Tumori Ligure, Genoa); Paolo Contiero, Giovanna Tagliabue (Registro Tumori Lombardia – Provincia di Varese); Maria Elisa Artioli, Katia Valla (Registro Tumori della Provincia di Modena); Vincenzo De Lisi, Lidia Serventi (Registro Tumori della Provincia di Parma); Francesco La Rosa, Fabrizio Stracci (Registro Tumori Umbro); Stefano Ferretti, Anna Rita Lombardi (Registro Tumori della Provincia di Ferrara); Massimiliano Oggiano, Amelia Sechi (Registro Tumori della Provincia di Sassari); Silva Franchini, Maria Gentilini (Registro Tumori della Provincia di Trento); Ettore Conti, Valerio Ramazzotti (Registro Tumori di Popolazione della Provincia di Latina); Adriano Giacomin, Lucia Preto (Registro Tumori della Provincia di Biella); Gianni Vicario, Loris Zanier (Registro dei Tumori del Friuli-Venezia Giulia); Francesco Bellù, Fabio Vittadello (Registro Tumori dell'Alto Adige); Susanna Vitarelli, Franco Pannelli (Registro Tumori Provincia di Macerata, Camerino); Rosario Tumino, Lorenzo Gafà (Registro Tumori Ragusa); Maurizio Montella, Mario Fusco (Registro Tumori della Provincia di Napoli); Stefano Boros, Patrizio Pezzotti (Istituto Superiore di Sanità, Roma).
